# Ribosomal protein genes in post-mortem cortical tissue and iPSC-derived neural progenitor cells are commonly upregulated in expression in autism

**DOI:** 10.1038/s41380-020-0773-x

**Published:** 2020-05-13

**Authors:** Michael V. Lombardo

**Affiliations:** 1 Laboratory for Autism and Neurodevelopmental Disorders, Center for Neuroscience and Cognitive Systems @UniTn, Istituto Italiano di Tecnologia, Corso Bettini, 31, 38068 Rovereto, Italy; 2 Department of Psychiatry, Autism Research Centre, University of Cambridge, Cambridge, UK

**Keywords:** Neuroscience, Autism spectrum disorders

## To the Editor

**Fig. 1:**
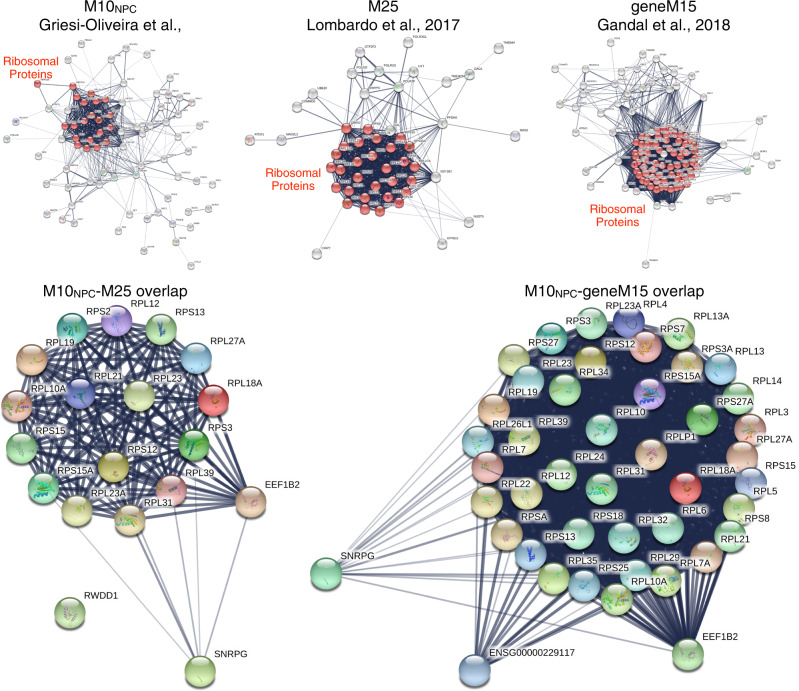
Graphs showing protein–protein interactions from STRING (https://string-db.org) for genes within co-expression modules M_NPC_10-blue from Griesi-Oliveira et al. [1] (top left), M25 from Lombardo et al. [4] (top middle), and geneM15 from Gandal et al. [5] (top right). Ribosomal proteins are highlighted in red. In the bottom left are genes that overlap between M_NPC_10-blue and M25, while on the bottom right are genes that overlap between M_NPC_10-blue and geneM15.

In a recent paper, Griesi-Oliveira et al. [1] identified a gene co-expression module in induced pluripotent stem cell (iPSC)-derived neural progenitor cells (NPCs) that is upregulated in expression in autism. The authors also show proteomic evidence to suggest that these earlier disruptions in translation in NPCs may lead to dysregulation of gene expression relevant to synaptic processes in neurons. These are valuable insights to contribute to the literature, particularly because iPSC models can be a useful model of early prenatal periods of development. Griesi-Oliveira et al. [1] also attempted to assess how similar this kind of transcriptomic dysregulation is to previously reported studies using gene expression data in post-mortem cortical tissue. As the authors rightly note, the papers they utilized for this analysis [2, 3] did not themselves report any co-expression modules with differential module eigengene expression in modules enriched for translation processes. However, in our recent paper [4], we re-analyzed data from these studies [2, 3] and indeed identified two consensus co-expression modules enriched in translation processes (M1 and M25) that were replicably upregulated in autism in both datasets. When we examine whether M_NPC_10-blue heavily overlaps with these translation-enriched modules, we indeed find high overlap with the translation-enriched M25 co-expression module (odds ratio (OR) = 15.52, *p* = 1.24e − 14). Evidence from a later study by Gandal et al. [5] also showed the presence of a co-expression module (geneM15) that is localized to the ribosome and enriched in excitatory neuronal cell types. This autism-upregulated geneM15 module from post-mortem cortical tissue highly overlaps with M_NPC_10-blue (OR = 19.10, *p* = 3.26e − 34) and the M25 module from our study (OR = 140.27, *p* = 1.09e − 54). Nearly all of the overlapping genes in M_NPC_10-blue and Lombardo et al.’s [4] M25 and Gandal et al.’s [5] geneM15 are ribosomal protein genes (e.g., *RPL* and *RPS*). See Fig. 1 for a graphical depiction of these gene sets and the overlap. The data and code that reproduces these results can be found here: https://github.com/mvlombardo/ipsc_translation. Thus, rather than there being no prior evidence of upregulated translation co-expression modules in post-mortem cortical tissue, there is such evidence from multiple studies [4, 5], as well as evidence to support that many of the same genes in M_NPC_10-blue are also those found within those translation-enriched co-expression modules from post-mortem cortical tissue.

A second point of interest is that our prior paper makes a statement about how the cortical transcriptome is hierarchically disorganized in autism [4]. Evidence to support this comes from the result that eigengenes from upregulated and downregulated co-expression modules are highly correlated, with increases in the expression of translation modules being associated with decreased expression of synaptic-enriched modules. In addition, dysregulated co-expression modules show a high degree of physical interactions at the protein level—that is, translation-enriched modules like M25 show a high level of protein–protein interactions with proteins from genes from synaptic-enriched co-expression modules. Thus, the statement that upregulated M_NPC_10-blue might be linked to dysregulation of neuronal co-expression modules in this iPSC dataset is indeed foreshadowed and predicted by our observations of this same kind of emergent disorganization across co-expression modules observed in post-mortem cortical tissue.

Finally, it is worth discussing the potential importance of these upregulated co-expression modules enriched in translation processes with respect to evidence already in the literature. The authors have cited that protein synthesis is a cellular process of interest in autism. Indeed, translational control has been of broad long-standing interest in autism research as many of the earliest known single gene mutations with high penetrance for autism are those that affect translational control (e.g., *FMR1*, *TSC2*, *PTEN*) [6]. These genes, along with others (e.g., *EIF4E*, *UBE3A*, *NF1*, *MECP2*, *SHANK3*), all affect AKT-PI3K-mTOR signaling pathways [7–11] and these pathways are known to be affected in idiopathic cases of autism [12, 13]. While these are important prior starting points for talking about protein synthesis in autism, there is a missed opportunity here to expand beyond this evidence. For example, the M_NPC_10-blue co-expression module has a large number of genes coding for 40S and 60S ribosomal subunits (*RPL* and *RPS* genes) and these genes are key members of our previously reported M25-upregulated translation-enriched co-expression module [4] as well as the autism-upregulated geneM15 module from Gandal et al. [5]. These genes as well as the ribosome cellular component itself are largely not focused on in the literature. The evidence from our paper [4] and others [14], as well as this new evidence from Griesi-Oliveira et al. [1] should underscore the idea that future work should focus more heavily on this type of translation-relevant biology. Furthermore, such upregulation of translation-relevant biology (e.g., genes for ribosomal proteins) are known to be similarly upregulated in maternal immune activation [15] and in non-neural cell types such as blood leukocytes from patients with autism [16, 17]. Finally, similar co-expression modules identified in blood leukocytes are associated with functional neural phenotypes relevant to receptive speech processing in autism with poor language outcome [18].

These insights raise questions for how future work might expand on the relevance of upregulated ribosomal protein genes in brain development in autism? Here I suggest a couple of routes that could be examined. First, the upregulated expression of ribosomal protein genes could suggest that ribosome biogenesis is enhanced, and thus protein synthesis is also enhanced. Therefore, examination of ribosome biogenesis could be an important avenue of future work in autism, particularly if enhanced ribosome biogenesis affects specific cell types (e.g., excitatory neurons, radial glia). Second, enhanced protein synthesis—be it through enhanced ribosome biogenesis or other known autism-associated mechanisms (e.g., *FMR1*, *TSC2*, *PTEN*, mTOR signaling)—could lead to enhanced cell proliferation. It is also known that cell proliferation and protein synthesis are tightly linked [19], and in cancer research, many mechanisms relevant to suppressing or enhancing cell growth and proliferation affect ribosome biogenesis and protein synthesis [20]. This route may be relevant for examining molecular mechanisms that could underlie accelerated early brain overgrowth in autism [11, 21] and/or subtypes of patients with much larger brains [22]. Given the insights from Griesi-Oliveira et al. [1] for showing this process is already upregulated in NPCs, this avenue could be interesting from the standpoint that specific types of progenitor cells (e.g., ventricular or outer radial glia) in very early fetal brain development are implicated in the expansion of cortical surface area [23, 24]. Third, ribosomal proteins can have non-ribosomal functions, such as involvement in the immune system [20]. These functions can emerge because steps in ribosome biogenesis can be disturbed (i.e. ribosomal stress) and lead to an accumulation of ribosome-free ribosomal proteins in the cell. Whether these extra-ribosome functions are a factor in autism is unknown, but this could be another route for how enhanced ribosomal proteins could potentially affect autistic patients.

In conclusion, the discoveries of Griesi-Oliveria et al. [1] are important and highlight the idea that upregulated translation processes and their interaction with other important biological processes taking place at the synapse be prioritized as a fruitful area for future discoveries. While translational control is itself an area of much interest in autism [6–10], there is likely more to this topic than currently understood and more emphasis could also be placed on the importance of ribosomal proteins.
